# 2488. Healthcare-associated infections in acute care hospitals in Greece, results of the third Point Prevalence Survey, April – December 2022

**DOI:** 10.1093/ofid/ofad500.2106

**Published:** 2023-11-27

**Authors:** Konstantinos Palaiopanos, Dimitra Krystallaki, Kassiani Mellou, Kassiani Mellou, Antonios Maragkos, Lida Politi, Dimitrios Paraskevis, Sotirios Tsiodras, Theoklis Zaoutis

**Affiliations:** National Public Health Organisation (EODY), Athens, Greece, Athens, Attiki, Greece; National Public Health Organisation (EODY), Athens, Greece, Athens, Attiki, Greece; Hellenic National Public Health Organization, Athens, Attiki, Greece; Hellenic National Public Health Organization, Athens, Attiki, Greece; National Public Health Organisation (EODY), Athens, Greece, Athens, Attiki, Greece; National Public Health Organisation (EODY), Athens, Greece, Athens, Attiki, Greece; National Public Health Organisation (EODY), Athens, Greece, Athens, Attiki, Greece; 4th Department of Internal Medicine, University General Hospital Attikon, Medical School, National and Kapodistrian University of Athens, 12462 Athens, Greece, Athens, Attiki, Greece; Hellenic National Public Health Organization, Athens, Attiki, Greece

## Abstract

**Background:**

The burden of healthcare-associated infections (HAIs) in European acute care hospitals is estimated through periodic Point Prevalence Studies (PPS) coordinated by European Centre for Disease Prevention and Control (ECDC). In 2016, Greece had the highest HAI prevalence among European countries (10%). The current PPS aimed to estimate the prevalence of HAIs among inpatients, especially in the context of COVID-19 pandemic.

**Methods:**

A cross-sectional study was conducted between April and December 2022, according to ECDC PPS protocol v.6.1, in a representative sample of 50 Greek hospitals across all country regions. Patients admitted in wards before 8.00 am of the study day were screened for inclusion. Infection control teams collected hospital characteristics (e.g., total beds, infection control markers), wards (specialty, bed occupancy) and active HAIs (type, origin, microbiological results). Active HAIs included present HAIs or past HAIs that are still being treated. ECDC HelicsWin.Net software v.4.4.0, was used for data entry and analysis.

**Results:**

Overall, 12.1% inpatients (1,175 of a total 9,707) had at least one active HAI. Among the 1,408 HAIs, the most commonly observed were lower respiratory tract infections (28.9%), bloodstream infections (20%), among which 4.6% were catheter-related, and urinary tract infections (13.1%), as shown in table 1. HAI prevalence was higher in patients of intensive care units (45.7%), compared to medical (13.5%) and surgical ones (8.2%).

Laboratory data were available for 58.9% of HAIs and 1,259 microorganisms were identified. *Klebsiella* species (20.5%), *Acinetobacter* species (12.8%), *P. aeruginosa* (10.2%), *Candida* species (7.9%) and *S. aureus* (6.3%) were frequent isolates. Antimicrobial susceptibility patterns varied depending on the pathogen (table 2).

**Figure:**
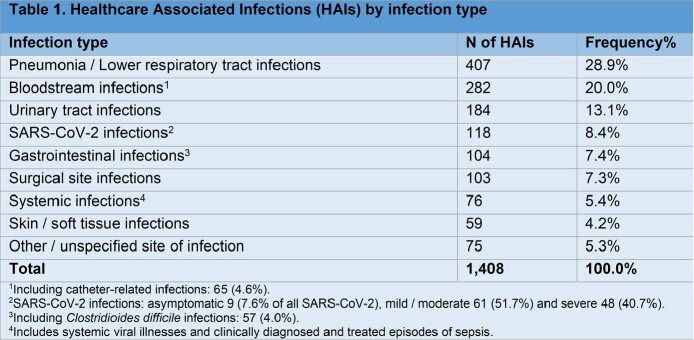

**Figure:**
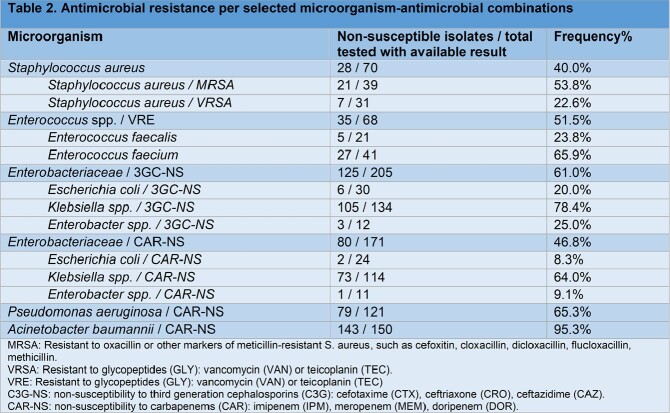

**Conclusion:**

This study shows the increased burden of HAIs among inpatients and the emergence of difficult-to-treat pathogens in Greek hospitals. Subsequent prevalence studies will provide additional evidence regarding the trend in HAI prevalence among inpatients and support the identification of modifiable practices. The invigoration of infection control and antimicrobial stewardship programs in these settings is essential.

**Disclosures:**

**All Authors**: No reported disclosures

